# Epidemiology of *Helicobacter pylori* and CagA-Positive Infections and Global Variations in Gastric Cancer

**DOI:** 10.3390/toxins10040163

**Published:** 2018-04-19

**Authors:** Jin Young Park, David Forman, Langgeng Agung Waskito, Yoshio Yamaoka, Jean E. Crabtree

**Affiliations:** 1 International Agency for Research on Cancer, 69372 Lyon, France; parkjy@iarc.fr (J.Y.P.); FormanD@visitors.iarc.fr (D.F.); 2 Institute of Tropical Disease, Universitas Airlangga, Surabaya 60113, Indonesia; langgengaw@gmail.com; 3 Department of Environmental and Preventive Medicine, Faculty of Medicine, Oita University, Yufu, Oita 879-5503, Japan; yyamaoka@oita-u.ac.jp; 4 Department of Medicine-Gastroenterology, Michael E. DeBakey Veterans Affairs Medical Center and Baylor College of Medicine, Houston, TX 77030, USA; 5 Leeds Institute Biomedical and Clinical Sciences, Wellcome Trust Brenner Building, St. James’s University Hospital, University of Leeds, Leeds LS9 7TF, UK

**Keywords:** *Helicobacter pylori*, gastric cancer, CagA, epidemiology, randomised controlled trial

## Abstract

Gastric cancer is a major health burden and is the fifth most common malignancy and the third most common cause of death from cancer worldwide. Development of gastric cancer involves several aspects, including host genetics, environmental factors, and *Helicobacter pylori* infection. There is increasing evidence from epidemiological studies of the association of *H. pylori* infection and specific virulence factors with gastric cancer. Studies in animal models indicate *H. pylori* is a primary factor in the development of gastric cancer. One major virulence factor in *H. pylori* is the cytotoxin-associated gene A (*cagA*), which encodes the CagA protein in the *cag* pathogenicity island (*cag* PAI). Meta-analysis of studies investigating CagA seropositivity irrespective of *H. pylori* status identified that CagA seropositivity increases the risk of gastric cancer (OR = 2.87, 95% CI: 1.95–4.22) relative to the risk of *H. pylori* infection alone (OR = 2.31, 95% CI: 1.58–3.39). Eradicating *H. pylori* is a strategy for reducing gastric cancer incidence. A meta-analysis of six randomised controlled trials (RCTs) suggests that searching for and eradicating *H. pylori* infection reduces the subsequent incidence of gastric cancer with a pooled relative risk of 0.66 (95% CI: 0.46–0.95). The introduction in regions of high gastric cancer incidence of population-based *H. pylori* screening and treatment programmes, with a scientifically valid assessment of programme processes, feasibility, effectiveness and possible adverse consequences, would impact the incidence of *H. pylori*-induced gastric cancer. Given the recent molecular understanding of the oncogenic role of CagA, targeting *H. pylori* screening and treatment programmes in populations with a high prevalence of *H. pylori* CagA-positive strains, particularly the more oncogenic East Asian *H. pylori* CagA strains, may be worth further investigation to optimise the benefits of such strategies.

## 1. Introduction: The Global Burden of Gastric Cancer

**Figure 1:**
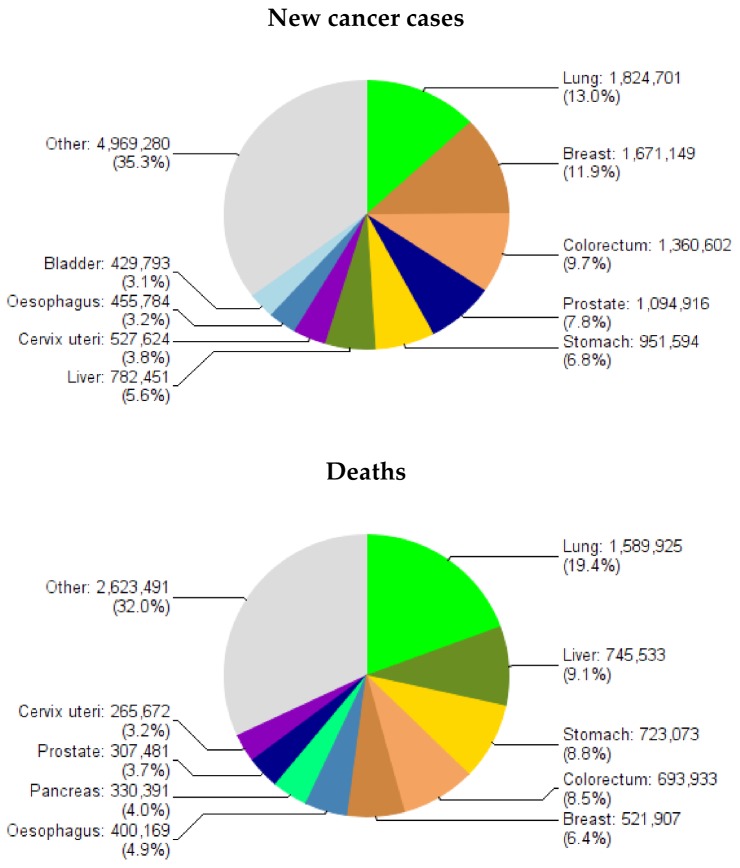
Estimated frequency of new cancer cases and deaths Worldwide in 2012, both sexes combined (Source: GLOBOCAN 2012, adapted from [1], 2013, International Agency for Research on Cancer).

**Figure 2:**
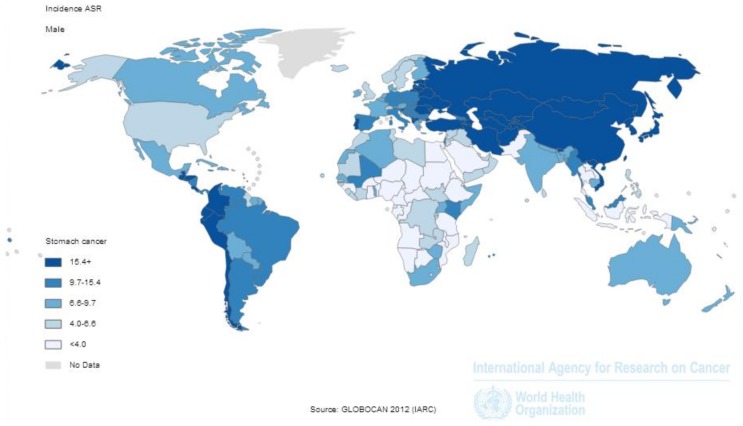
Estimated age-standardised (world) incidence rates of gastric cancer per 100,000 by country among males in 2012 (Source: GLOBOCAN 2012, adapted from [1], 2013, International Agency for Research on Cancer).

**Figure 3:**
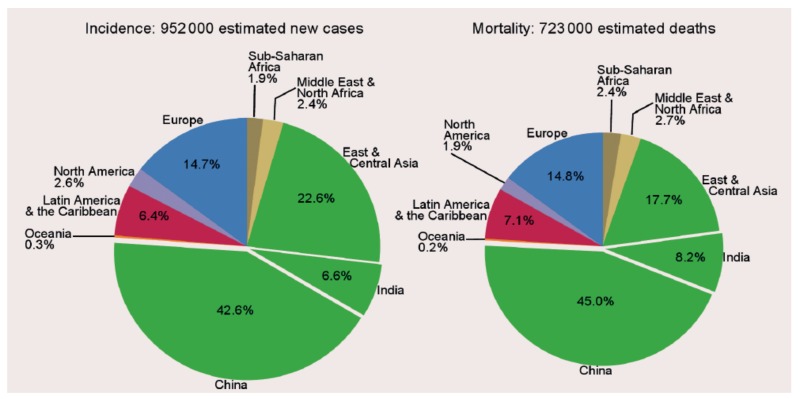
Gastric cancer: number of new cases and deaths with proportions by major regions (both genders combined) in 2012 (Source: GLOBOCAN 2012, adapted from [1], 2013, International Agency for Research on Cancer).

Gastric cancer is the fifth most common malignancy in the world (after lung, breast, colorectal and prostate cancers). Globally, nearly one million (952,000) new cases and 723,000 deaths were estimated to have occurred from this disease in 2012 [1]. It accounts for 7% of new cancer cases and 9% of all cancer deaths worldwide (Figure 1) [1]. The distribution map for the incidence of male gastric cancer shows wide variation between geographic regions with the highest rates observed in Asia, Central and South America and Eastern Europe (Figure 2). The pattern of distribution for females is almost identical to that for males although female rates are usually around half those in males (data not shown, [1]). Over 70% of new gastric cancer cases that occurred in 2012 were in less developed regions of the world with Asia contributing approximately 72% of the global burden and almost half of the cases in the world occurring in China (Figure 3) [1].

Using the high-quality cancer registry data from the Cancer Incidence in Five Continents Volume XI (CI5 XI) [2], Table 1 identifies populations with some of the highest and lowest observed male gastric cancer rates in the world for the period 2008–2012. The magnitude of the difference is over 14-fold as seen by comparing the incidence rates in populations within China, the Republic of Korea and Japan (at or over 70 per 100,000) with those in several African, Asian and USA populations, which did not exceed 5 per 100,000. Overall, global patterns of incidence and mortality are very similar to each other because prognosis following a diagnosis of gastric cancer is usually poor [3]. However, mortality rates in Japan and the Republic of Korea are, in comparison with other populations, considerably lower than the corresponding incidence rates. This likely reflects the impact of screening and early diagnosis in these countries.

**Figure 4:**
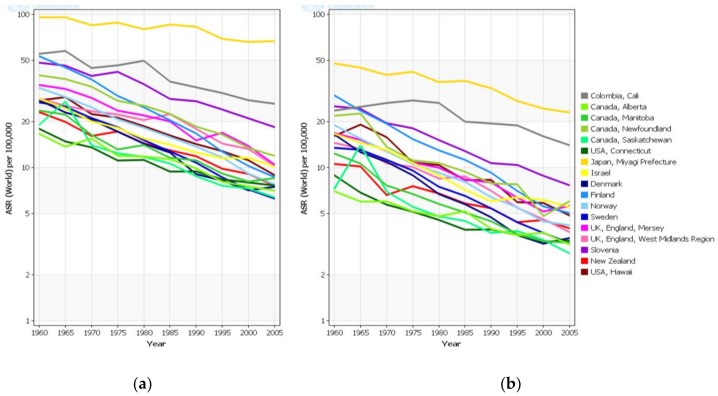
Gastric cancer: age-standardised (world) incidence rates by year for cancer registries in CI5 I-X (Source: Cancer Incidence in Five Continents, CI5plus, adapted from [4], 2014, International Agency for Research on Cancer). (**a**) Males (all ages); (**b**) Females (all ages).

Figure 4, based on the first ten volumes of CI5 [4], shows the trends in age-standardised incidence rates for males and females for selected populations covering the period 1960–2005. Over the 45-year period, incidence rates have steadily declined in nearly all populations with similar trends in both males and females. The downward trends can be observed regardless of the level of background risk and, even in areas with historically very high gastric cancer rates such as Japan and Colombia, decreasing trends can be observed in recent years. Despite the global decline in incidence rates over many years, the absolute burden of gastric cancer (number of cases diagnosed) has remained high as a result of population growth and ageing. Indeed, even if gastric cancer rates continue to decline at around the present level of approximately −2% per annum, the absolute burden is likely to remain static for the next 10–20 years because of these demographic factors (Table 2). 

In general, the distribution of gastric cancer by subsite of the stomach has not been well characterised in international comparative datasets. A recent estimate of the proportion of cardia and non-cardia gastric cancers using the same data sources as above indicates that approximately 27% of gastric cancers (260,000 of the 952,000 worldwide) arise in the cardia region of the stomach with the remaining 73% (691,000) defined as non-cardia [5]. The geographic variation of the two subsites is broadly similar with the highest rates estimated to occur in Asia. However, whereas most populations show higher rates of non-cardia cancer, this is not consistently the case and in some countries, such as Australia, the USA and the UK, rates are similar to each other [5].

## 2. The Association of Gastric Cancer with *Helicobacter pylori* Infection

### 2.1. Evaluation by International Agency for Research on Cancer (IARC) Working Groups

*Helicobacter pylori* was initially considered and classified as carcinogenic to humans (Group I carcinogen) by an International Agency for Research on Cancer (IARC) Working Group in 1994 based on the results from a small number of studies then available (four cohort and nine case–control) that considered gastric carcinoma [6]. In 2009, a new Working Group reviewed considerably more data which had become available since the previous evaluation and reconfirmed that chronic infection with *H. pylori* is a Group 1 carcinogen with sufficient evidence of causing non-cardia gastric carcinoma and low-grade B-cell gastric MALT lymphoma. 

In 2009, a new Working Group reviewed considerably more data which had become available since the previous evaluation and reconfirmed that chronic infection with *H. pylori* is a Group 1 carcinogen with sufficient evidence of causing non-cardia gastric carcinoma and low-grade B-cell gastric MALT lymphoma [7].

### 2.2. Quantification of Risk—Comparison of Western Blot and Serology Data

The 2009 Working Group noted a substantial increase in the estimated odds ratios (ORs) for the association between *H. pylori* infection and non-cardia gastric carcinoma in studies using Western blotting assays compared with enzyme-linked immunosorbent assay (ELISA) for the analysis of serum IgG responses to *H. pylori.* Using ELISAs, statistically significant ORs varied widely from 1.6 to 7.9, whilst studies using Western blotting analysis to determine infection status reported an increase in the ORs up to 10.6 [8] or 17.8 [9]. This observation was later re-confirmed in a nested case–control study of the European Prospective Investigation into Cancer and Nutrition study (Eurgast-EPIC), which showed that the magnitude of the non-cardia gastric cancer risk associated with *H. pylori* infection in IgG serological studies was more than three-fold higher by Western blot assay than by ELISA (OR was 21.4 and 6.8 by immunoblot and ELISA, respectively) [10]. Earlier Western blotting analysis of both IgG serological and gastric mucosal IgA responses to *H. pylori* have also been investigated. Resected non-involved gastric mucosa of patients with gastric cancer was cultured in vitro to investigate mucosal IgA responses to *H. pylori.* Interestingly, several *H. pylori* IgG serological negative patients with gastric cancer secreted mucosal IgA against multiple *H. pylori* proteins indicative of a previous positive *H. pylori* status [11].

### 2.3. Other Evidence: Animal Studies/Mechanistic Understanding

**Figure 5:**
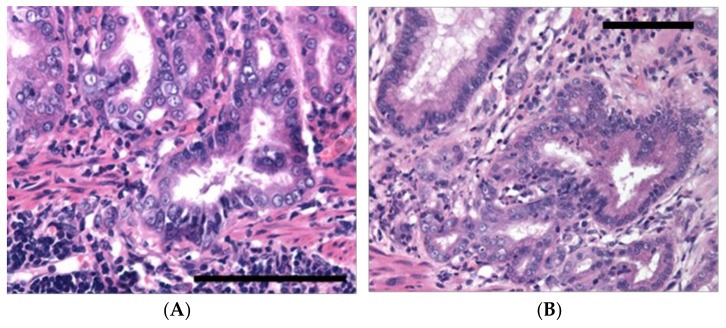
Histopathology of gastric mucosa of Mongolian gerbils infected with 3GX Chinese strain of *Helicobacter pylori*. Adapted from the Journal of Pathology [28], 2006, Wiley. Haematoxylin and eosin-stained sections of corpus gastric mucosa of Mongolian gerbils 30 weeks post-infection with *H. pylori* strain 3GX. (**A**) High-grade dysplasia involving intramucosal and herniating glands. Bar = 50 μm; (**B**) Breakup of the architecture of intramucosal glands with isolated clusters of epithelial cells in the lamina propria indicative of intramucosal carcinoma. Bar = 100 μm.

The IARC Working Group evaluation also considered experimental evidence in Mongolian gerbils and mice and concluded that there is sufficient evidence for the carcinogenicity of infection with *H. pylori* in experimental rodent models [7]. *H. pylori*-infected Mongolian gerbils developed gastric adenocarcinoma in the majority of studies [12,13,14,15,16,17,18] (Figure 5). However, in inbred mice with *H. pylori* infection, gastritis was evident whilst gastric adenocarcinoma did not develop [19,20,21]. In several transgenic mice, such as TGF-β deficient [22], p27 deficient [23] and Trefoil factor family 2 (TFF2) deficient [24], infection with *H. pylori* showed synergistic effects in gastric cancer development [7]. In addition, *H. pylori* infection of transgenic mice overexpressing gastrin could induce gastric adenocarcinoma [25], while gastric carcinogenesis was inhibited when *H. pylori* eradication therapy was introduced [26]. *H. pylori* eradication in mice deficient in p27 similarly reversed premalignant lesions [27].

**Figure 6:**
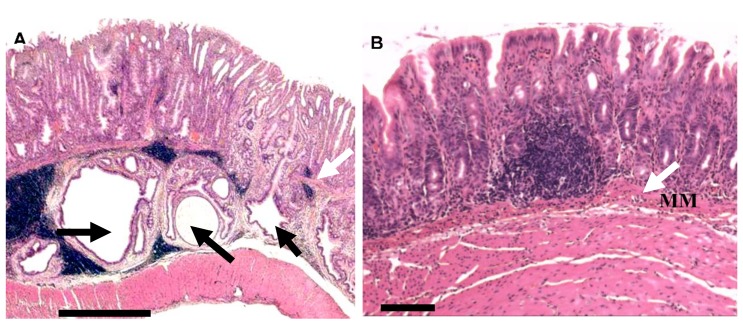
Pathology of gastric mucosa of *H. pylori*-infected Mongolian gerbils treated with an epidermal growth factor receptor (EGFR) inhibitor diet and control diet. Haematoxylin and eosin-stained sections of gastric antral mucosa of Mongolian gerbils 38 weeks post-infection with *H. pylori* SS1 strain. (**A**) *H. pylori*-infected gerbil on the control diet. Bar = 800 µm. White arrows indicate the muscularis mucosa, which is discontinuous on the right-hand side, and the three black arrows indicate large submucosal herniations. The right-hand side herniation is sectioned longitudinally reaching the gastric lumen. (**B**) Antral mucosa of *H. pylori*-infected gerbil on the EKB-569 diet lack submucosal herniations but have an intact muscularis mucosa (MM) indicated by the white arrow. Bar = 100 μm. Reproduced with permission of Pathogens [32], 2013, MDPI.

The Mongolian gerbil model, in which *H. pylori*-induced gastric pathology progresses from antral gastritis to pan gastritis with corpus atrophy in a similar manner to human gastric pathology, in *H. pylori* infected individuals [28,29] has also been used to assess the effects of *H. pylori* eradication on the development of gastric cancer [17]. In addition, following the identification that *H. pylori* transactivates the epidermal growth factor receptor (EGFR) in gastric epithelial cells [30,31], the therapeutic effects of EGFR inhibitors on *H. pylori*-induced pathology have been examined in gerbil and murine models. Treatment of *H. pylori*-infected gerbils with the selective EGFR inhibitor EKB-569 for 32 weeks significantly reduced corpus atrophy, mucous metaplasia, submucosal herniations (Figure 6) and markedly reduced gastric epithelial cell proliferation to apoptosis ratios [32]. The submucosal herniations are thus a consequence of changes in epithelial kinetics and dependent on EGFR transactivation. Recent studies have also indicated that short-term treatment with the EGFR inhibitor gefitinib reduced DNA damage and gastric inflammation in *H. pylori*-infected mice and gerbils [33]. These therapeutic studies have shed light on important mechanisms by which *H. pylori* eradication therapy and the targeting of key pro-carcinogenic *H. pylori-*induced signalling pathways, such as EGFR transactivation, may protect against *H. pylori* gastric carcinogenesis.

## 3. *H. pylori* Infection: Geographic and Time Trends

*H. pylori* infection is known to be common with a global prevalence of over 50%; however, there is substantial country-to-country variation. A recent systematic review of 37 studies with national coverage in 22 countries reported a prevalence of approximately 70% or higher around age 60 years in Central and South America and Asia in the late 1990s and early 2000s, with a decreasing trend in most countries where data are available for different time periods [34]. The prevalence of *H. pylori* infection was at least two-fold higher in countries with high gastric cancer incidence, both in young adults and in older participants, compared with countries with a lower background risk [34].

A combination of the high prevalence of *H. pylori* infection and the subsequent high OR for the association with gastric cancer results in a high attributable risk. Based on the 952,000 new gastric cancers diagnosed each year globally, it has recently been estimated that 753,000 (79%) of these could be attributed to *H. pylori* infection with a further 13,000 gastric lymphomas also likely to be *H. pylori*-associated [35]. These estimates were based on a prevalence of *H. pylori* infection in gastric non-cardia cancer cases of 94.6% and a relative risk of 17.0. These estimates indicate that *H. pylori* infection should be considered the primary risk factor for gastric cancer, and it has been proposed that infection might be considered “a (close to) necessary cause of non-cardia gastric cancer” [36].

## 4. *H. pylori* Strain Variation and Gastric Cancer Risk

### 4.1. CagA and the Cag Pathogenicity Island 

Early studies found that not all *H. pylori* strains expressed a protein of circa 120 K and mucosal IgA responses to this protein are significantly associated with peptic ulcer disease and with active gastritis characterised by increased gastric epithelial neutrophil infiltration [37]. The 120 K protein, subsequently called CagA, was identified as a product of the multigene *cag* pathogenicity island (*cag* PAI), a type IV secretory system [38,39]. CagA, which is considered an oncoprotein, is translocated into gastric epithelial cells by the type IV secretory system of the pathogen, inducing multiple signalling cascades [40,41]. Other reviews in this series detail the epithelial intracellular signalling responses induced by CagA and its role in disease pathogenesis [42,43]. The *cag* PAI also translocates peptidoglycan breakdown products into gastric epithelial cells, which activate intracellular NOD1 and NFκB [44,45], thus contributing to enhanced C-X-C chemokine responses and active gastritis in *cag* PAI-positive infections [37,44,45,46].

Recent studies have reported that in addition to NOD-1 activation, bacterial heptose-1,7-bisphosphate (HBP), a metabolic precursor of lipopolysaccharide biosynthesis, is also delivered via the *cag* type IV secretory system into epithelial cells [47,48]. Independently of NOD1, HBP activates cytosolic tumour necrosis factor receptor-associated factor (TRAF) interacting protein with forkhead-associated protein (TIFA), which results in early activation of NFκB and C-X-C chemokine responses. Thus, activation of epithelial NOD-1 and TIFA by two independent components of *H. pylori* delivered via the *cag* type IV secretory system triggers innate immune inflammatory responses. 

### 4.2. CagA Serology and Corpus Atrophic Gastritis

The CagA protein is immunodominant and is recognised immunologically early following infection with *H. pylori* CagA-positive strains by both gastric mucosal IgA and serum IgG responses [49]. With the availability of recombinant CagA [50,51], ELISA assays to determine CagA IgG seropositivity in the gastric cancer precursor condition corpus atrophy and non-cardia gastric cancer has been the focus of many studies. Several studies have examined the association of CagA IgG seropositivity with corpus atrophic gastritis diagnosed pathologically following endoscopic investigation [22,23,24,25,52,53,54,55]. Other studies have been undertaken in non-endoscoped subjects where the ratio of serum pepsinogen A and pepsinogen C, an established marker of gastric corpus atrophy, has been measured in the absence of histological diagnosis [56,57].

Followup endoscopic studies on corpus atrophy-negative *H. pylori-*positive patients after a time span of 11 years reported 62% of CagA IgG seropositive subjects (*n* = 24) had developed atrophic gastritis versus 32% of *H. pylori*-positive CagA IgG seronegative subjects (*n* = 34) (OR = 3.48; 95% CI: 1.02–12.18) [52]. In dyspeptic *H. pylori* positive patients (*n* = 267) undergoing endoscopy in 14 European countries, the combined presence of IgG antibodies to CagA and the *H. pylori* vacuolating cytotoxin A (VacA) was also significantly associated with atrophic corpus gastritis (OR = 3.09, 95% CI: 1.26–7.56) [55]. Infection with CagA-positive strains is thus associated with increased risk of developing atrophic corpus gastritis compared to CagA-negative *H. pylori*-positive subjects. Supporting evidence comes from animal studies. Infection of Mongolian gerbils with a wild-type *H. pylori* strain with a functional *cag* type IV secretory system and isogenic mutant strains lacking *cag*A or *cag*Y indicate that a functional *cag* PAI facilitates corpus colonisation and corpus atrophy [58].

The Eurogast study included 17 centres in 13 counties with 2850 randomly selected subjects in whom serum pepsinogen A/C ratio was determined as a marker of corpus atrophy [56]. Assays for CagA IgG seropositivity in all 17 centres in the Eurogast study found that *H. pylori* IgG seropositive subjects who were CagA seropositive had significantly lower pepsinogen A/C ratios than *H. pylori* seropositive subjects who were CagA seronegative. These studies reinforce the association of CagA-positive strains with corpus atrophy in multiple European countries [56].

The majority of studies on the association of CagA-positive *H. pylori* infection and corpus atrophy have to date been undertaken in developed countries. In developing countries, factors that may beneficially modulate *H. pylori*-induced pathology are co-infection with helminths, which increase T regulatory cells and polarise inflammation to Th2 responses [59]. Clinical evidence in *H. pylori*-infected Chinese subjects in the Dongting Lake area indicate that co-infection with the digenean helminth *Schistosoma japonicum* significantly reduces gastric atrophy in *H. pylori*-infected subjects as determined by increased pepsinogen A:pepsinogen C ratio in CagA seronegative subjects [57]. Further studies from developing countries on the effects of co-infections and their impact on the progression of CagA-positive *H. pylori*-associated corpus gastritis are required.

### 4.3. CagA Seropositivity and Risk of Non-Cardia Gastric Cancer

The marked global variation in both the incidence of gastric cancer ([1,2,3], Table 1) and the global variability in the frequency of *cag* PAI-positive *H. pylori* strains [60] are important factors to consider when assessing the relative risk of infection with CagA-positive *H. pylori* strains and distal gastric cancer. Functional differences in CagA tyrosine phosphorylation sites between Eastern and Western CagA-positive *H. pylori* strains are evident [61,62]. Recent studies indicate that CagA EPIYA-D in East Asian strains binds to the pro-oncogenic SHP2 phosphatase 100-fold more strongly than CagA EPIYA-C in Western *H. pylori* strains and also stimulates stronger cellular Ras–ERK activation [63]. CagA IgG serological status has been evaluated by ELISA using recombinant CagA in several gastric cancer case–control and cross-sectional studies to determine whether CagA IgG seropositivity is associated with an increased incidence of non-cardia gastric cancer relative to CagA seronegative *H. pylori*-positive individuals and age-matched uninfected controls [64,65,66,67,68,69].

In two gastric cancer case–control studies [64,65] serum samples were available from 13–14 years before the diagnosis of gastric cancer. In a study of 261 Japanese American men with distal intestinal-type gastric cancer, *H. pylori*-positive CagA-negative subjects had an OR of 2.7 (95% CI: 1.3–5.6) for intestinal-type gastric cancer compared to uninfected controls [64]. Men who were both *H. pylori* and CagA seropositive however had an increased risk of intestinal-type gastric cancer with an OR of 4.1 (95% CI: 2.2–7.7) [64]. In the second study, 179 North American subjects infected with *H. pylori* who were CagA seropositive similarly had increased risk of gastric cancer (OR = 5.8, 95% CI: 2.6–13) relative to uninfected controls [65]. This increased risk was evident in both intestinal-type gastric cancer (OR = 5.1, 95% CI: 2.1–12.2) and diffuse-type gastric cancer (OR = 10.1, 95% CI: 2.2–47.4). An endoscopic study [66] in 81 indigenous Japanese diagnosed with gastric cancer and gender- and age-matched endoscopic controls reported that CagA seropositivity was significantly increased (*p* < 0.05) in patients with gastric cancer relative to non-cancer controls. The risk of gastric cancer with CagA seropositivity (OR = 1.93, 95% CI: 1.01–3.68) was lower than that in an earlier study [64] in emigrant Japanese men. The serum samples in the endoscopic study were taken at the time of diagnosis [66], in contrast to several years before diagnosis in the study of Nomura et al. [64]. Increasing gastric intestinal metaplasia with long-term *H. pylori* infection will decrease the density of *H. pylori* infection and thus CagA serological positivity. Furthermore, the high frequency of CagA-positive strains in Asia [60] may impact the assessment of the relative risk of gastric cancer.

A study in 103 younger (<40 years) Japanese patients with gastric cancer and age-matched controls found both *H. pylori*-positive CagA seronegative and *H. pylori*-positive CagA seropositive had an increased OR of gastric cancer [67]. The majority of these young Japanese patients had diffuse-type gastric cancer, the epidemiology of which differs from intestinal-type gastric cancer. In contrast to the studies in Japan, a European study in nine countries found that CagA positivity was associated with a 6.5-fold OR for non-cardia gastric cancer (OR = 6.5, 95% CI: 3.3–12.6) compared to *H. pylori*-positive CagA seronegative subjects [68].

Ethnic differences in CagA antibody responses in non-cardia gastric cancer in two Brazilian case–control groups have also been reported [69]. One group consisted of non-Japanese Brazilians (178 cancers, 178 controls) and the other group consisted of Japanese Brazilians (80 cancers, 160 controls). The study used a commercial European CagA ELISA. Higher CagA antibody titres were observed in non-Japanese Brazilians than Japanese Brazilians, probably reflecting differences in Asian and Western CagA [60,61,62,63]. The OR of non-cardia gastric cancer in CagA seropositives was higher in non-Japanese Brazilians (OR = 4.5, 95% CI: 2.6–7.8) than in Japanese Brazilians (OR = 2.1, 95% CI: 1.2–3.6) [69]). Interestingly, in both ethnic groups, the OR of non-cardia gastric cancer was highest in groups with low positive CagA antibody titres, which would be consistent with a reduced bacterial load in severe corpus atrophy. Similar observations were made by Suzuki et al. who found in a Japanese population that the relative risk (RR) of non-cardia gastric cancer in *H. pylori* seropositives with positive low CagA antibody titres was greater (RR = 3.9; *p* < 0.001) than that of subjects with high CagA antibody titres (RR = 2.0; *p* = 0.002) [70]. 

Meta-analysis of CagA serology determined in 10 non-cardia gastric cancer case–control studies with age- and gender-matched controls reported CagA seropositivity irrespective of *H. pylori* status was 62.8% in cases (*n* = 1707) and 37.5% in controls (*n* = 2124). CagA seropositivity increased the risk of gastric cancer (OR = 2.87, 95% CI: 1.95–4.22) relative to the risk of *H. pylori* infection alone (OR = 2.31, 95% CI: 1.58–3.39) [71]. A more recent meta-analysis of 10 gastric cancer case–control studies with 4325 patients in East Asian countries similarly identified that CagA seropositivity was also associated with increased risk of developing gastric cancer [72]. However, the OR in the meta-analysis in East Asian countries (OR = 1.81, 95% CI: 1.30–2.11) was lower than that in the meta-analysis of Huang et al. [71], which included Western populations.

### 4.4. Cholesterol Reduction Impacts on H. pylori-Induced Gastric Cancer Risk.

The use of certain pharmacological agents may also affect the outcomes of gastric cancer case–control studies investigating CagA seropositivity. The translocation of CagA and peptidoglycan breakdown products/ HBP into gastric epithelial cells via the *cag* PAI is key in triggering, respectively, oncogenic signalling pathways and inflammatory innate responses via NFkB activation (see Section 4.1). *H. pylori*-induced CagA translocation and induction of IL-8 in gastric epithelial cells can be reduced by cholesterol depletion [73,74], suggesting that long-term treatment with statins to reduce serum cholesterol levels could modify *H. pylori* signalling responses and pathogenesis. In a large case–control study of 19,728 Taiwanese with gastric cancer, and similar number of controls, previous long-term treatment with simvastatin was shown to significantly reduce the risk of gastric cancer (OR = 0.76, 95% CI: 0.70–0.83) after adjusting for *H. pylori* infection, gastric diseases, gastroesophageal reflux disease, gastric polyp, and gastritis. Those subjects with distal gastric cancer (*n* = 4539) who had received previous long-term treatment with simvastatin had a relative risk of 0.65 (95% CI: 0.57–0.75) of gastric cancer compared to controls who had not received previous long-term treatment with simvastatin [73]. These studies emphasise the importance of collecting pharmacological data in case–control studies investigating *H. pylori* and gastric cancer risk.

### 4.5. Lack of Association of CagA Seropositivity with Oesophageal Adenocarcinoma

Whilst most studies to date have focused on *H. pylori* CagA seropositivity and risk of distal gastric cancer, other important epidemiological studies have examined the association of CagA seropositivity with oesophageal adenocarcinoma (OAC) and oesophageal squamous cell carcinoma (OSCC). In contrast to gastric cancer, which is decreasing in many developed countries with the reduction in *H. pylori* infection, OAC is increasing. Ye et al. [75] in a population case–control study in Sweden examined CagA IgG seropositivity in 97 patients with OAC, 85 patients with OSCC, and 499 controls. CagA positivity was associated with a reduced risk of OAC (OR = 0.5; 95% CI: 0.3–0.8). In contrast, CagA antibody positivity and gastric atrophy were associated with increased OSCC (OR = 2.1, 95% CI: 1.1–4.0). Wu at al [76] in a population-based case–control study in Los Angeles examined the association of CagA seropositivity with distal gastric cancer (*n* = 127) and OAC (*n* = 80). Whilst finding a significant association of *H. pylori* and CagA seropositivity compared to *H. pylori* seronegativity with distal gastric cancer (OR = 2.2, 95% CI: 1.13–4.26), no association was evident between CagA seropositivity and OAC [76]. The above studies suggest that CagA is not associated with OAC, supporting the hypothesis that *cag*A+ strains may be protective by reducing OAC [77], potentially by modifying gastric physiological responses.

An interesting study examining retrospective cohorts of 61,548 unoperated patients with duodenal ulcers and 81,379 unoperated patients with gastric ulcers in the Swedish Inpatient Register surprisingly showed patients with duodenal ulcers had a 70% higher risk of OAC (standardised incidence ratio (SIR) = 1.7, 95% CI: 1.1–2.5) [78]. Gastric ulcers were unrelated to OAC (SIR = 1.1, 95% CI: 0.6–1.7) but were associated with an 80% increased risk of OSCC (SIR = 1.8, 95% CI: 1.4–2.3). In contrast, in patients with duodenal ulcers, there was a small excess with OSCC (SIR = 1.3, 95% CI: 0.96–1.8) [78]. Patients with duodenal ulcers have antral predominant gastritis and do not develop atrophic gastritis in the gastric corpus mucosa. The above differences in OAC and OSCC risk with *H. pylori* infection in subjects with duodenal or gastric ulcers likely reflect different patterns of *H. pylori* gastric colonisation, acid secretion and associated genetic variation in acid regulatory cytokines [28], as well as environmental factors such as smoking.

### 4.6. Microbiological Evidence Linking CagA/cag PAI Strains with Gastric Cancer

CagA has been recognised as an important factor in the development of gastric cancer in *H. pylori*-infected individuals. In a gerbil model, infection with a *cagA-*positive strain, but not the isogenic *cagA* mutant, resulted in development of gastric cancer [13]. Individuals infected with *cagA-*positive strains develop gastric cancer in Western populations [11,64,65]. However, in East Asian countries, most *H. pylori* strains express *cagA*; therefore, CagA status alone cannot explain the clinical outcomes [79]. The mosaicism of the CagA N-terminal repeat region on the residue Glu-Pro-Ile-Tyr-Ala (EPIYA) motif and its surrounding region categorise the EPIYA segment, known as EPIYA-A,-B and -C/-D, and can discriminate respectively Western-type CagA and East Asian-type CagA [80]. Epidemiological observations indicate that individuals infected with East Asian-type CagA *H. pylori* strains have a closer association with gastric mucosal atrophy and higher incidence of gastric cancer than Western-type CagA *H. pylori* strains [81,82,83]. This different cluster of CagA was also associated with a different cluster of the *cag* PAI, known as the Japanese cluster. Individuals infected with the Japanese cluster strains were reported to have higher atrophic gastritis scores than the Western type counterpart [84]. Epithelial translocated East Asian-type CagA also has a stronger binding to the host SHP2 domain, suggesting an increased signalling cascade in host epithelial cells [85].

### 4.7. Environmental Factors Regulating H. pylori CagA Expression

Apart from the internal mosaicism of CagA, which may reveal different activity of this oncogenic protein, environmental factors are also important in the regulation of CagA expression. Reported environmental factors that may be important are pH, salt and host serum iron concentrations [86,87,88]. The first observation of pH modifying *cagA* expression was undertaken using array studies. The *cagA* expression was significantly higher at pH 6.0 compared to pH 7.0 [86]; a similarly higher *cagA* expression was also observed at pH 4.0 [89]. In a recent study, differential RNA-seq was introduced to characterise the transcriptome expression in *H. pylori*. This study indicated that the expression of *cagA*, determined by the amount of cDNA, was two-fold higher under acid–stress conditions (pH 5.2) compared to a neutral environment (pH 7.0) [90]. However, the low pH has advantages to the host, reducing the survival of *H. pylori*, especially strains with a high number of EPIYA repeat regions [91].

High salt diets have been shown in epidemiological studies to be associated with an increased risk of gastric cancer. Intriguingly, recent studies indicated that a high salt diet modifies the expression of CagA in *H. pylori* [88]. High salt concentrations increased *H. pylori* CagA/*cagA* at both a protein and transcriptome level. RNA array analysis indicated *cagA* transcription had a two-fold increase in high salt concentrations [88]. Subsequent analysis using RT-qPCR methods also showed an increased *cagA* transcriptome [92]. Increasing expression of CagA was also observed by protein analysis. In addition, high salt concentrations mediated effectively the translocation process of CagA into AGS gastric epithelial cells [88]. High salt conditions also affected the expression of *cagA* in in vivo studies. In *cagA*-positive *H. pylori*-infected gerbils, a high salt diet induced higher *cagA* expression compared to *H. pylori*-infected gerbils on a regular diet. The gerbil group on a high salt diet infected with *cagA*-positive *H. pylori* strains had higher levels of inflammatory cytokines (IL-1, IL-6, IL-17, and gamma interferon [IFN-γ]), anti-inflammatory cytokines (IL-10), chemokines (KC, CCL12), and inducible nitric oxide synthase (iNOS). The higher expression of the *H. pylori* CagA oncogenic protein and the enhanced host immune response resulted in higher inflammation scores as well as a higher frequency of gastric carcinoma [92]. 

There is epidemiological evidence of a decreased risk of gastric cancer in subjects with a high iron status [93]. Iron has also recently been reported to have an association with CagA regulation. An animal model was used to examine the role of iron deficiency on CagA expression. Iron-depleted gerbils upregulated CagA expression 1.5 fold compared to expression in iron-replete gerbils [87]. Increased CagA expression was also concomitant with an increased assembly of the *cag* type IV secretion system, almost 2-fold higher, in nine visualised pili on strains from iron-replete versus 17 visualised pili on strains from iron-depleted gerbils. The translocation of CagA into gastric epithelial cells was also increased. Gerbils on iron-depleted diet infected with wild-type *H. pylori*, but not the *cagA*-negative isogenic mutant, were significantly quicker to develop dysplasia and adenocarcinoma (within 6 weeks of infection) than iron-replete gerbils [87]. In addition, clinically decreasing serum ferritin levels was inversely associated with premalignant lesions [87]. These data suggest CagA-positive infections and iron-depleted conditions have a significant role in the development of gastric cancer.

A subsequent study in iron-depleted gerbils indicated that *cagA*-negative *H. pylori* transform into the coccoid form, a non-viable *H. pylori* morphology, suggesting that CagA provides an advantage to the viability of *H. pylori* [94]. Interestingly, the hypervirulent strains, as a result of colonisation under iron-depleted conditions, had significantly decreased levels of CagA expression and epithelial translocation after passaging several times, suggesting the hypervirulent phenotype can be reversed [94]. These studies [87,94] demonstrate host iron stores can have an important impact on *H. pylori*-related gastro-duodenal diseases and suggest iron-deficient subjects may have increased risk of developing gastric cancer.

## 5. *H. pylori* Eradication for Gastric Cancer Prevention

### 5.1. Critical Evidence: Prospective Studies and Trials

The strong evidence for the carcinogenicity of chronic *H. pylori* infection suggests a clear path to preventing gastric cancer cases through eradication of the infection [95]. Population-based search-and-treat *H. pylori* programmes have, therefore, been proposed as a means to prevent gastric cancer [96], and international consensus and guidelines have included this strategy for gastric cancer prevention, especially in communities at high risk [97,98]. However, no country has yet implemented such programmes, partly due to lack of confidence in the effectiveness of *H. pylori* eradication in reducing gastric cancer and uncertainty about the possible harm of mass antibiotic treatment and its impact on gut microbiota [95].

The current scientific evidence that gastric cancer risk is reduced by *H. pylori* eradication is based on a small number of randomised controlled trials (RCT) of limited to moderate quality in healthy asymptomatic infected (mainly Asian) individuals [99]. A systematic review and meta-analysis of six RCTs suggest that searching for and eradicating *H. pylori* infection reduces the subsequent incidence of gastric cancer with a pooled relative risk of 0.66 (95% CI: 0.46–0.95) [99]. The authors noted that these data cannot necessarily be extrapolated to other populations as all except one study was conducted in East Asia [99].

A more recent systematic review and meta-analysis of 24 prospective studies also concluded that individuals receiving *H. pylori* eradication therapy had a lower incidence rate of gastric cancer than those without eradication therapy (a pooled rate ratio of 0.53 (95% CI: 0.44–0.64) after adjustment for baseline gastric cancer incidence) [100]. Subgroup analyses showed greater benefit among individuals after endoscopic resection of gastric cancers (pooled incidence rate ratio based on two RCTs and eight cohort studies, 0.46; 95% CI: 0.35–0.60) than among asymptomatic infected individuals (pooled incidence rate ratio based on six RCTs and eight cohort studies, 0.62; 95% CI: 0.49–0.79), however, with no statistical difference between the two [100]. This study also noted that the quantitative benefit of *H. pylori* eradication was greater in studies with higher baseline incidence rates of gastric cancer.

Despite the observed preventive effect of *H. pylori* eradication in gastric cancer, potential adverse consequences of mass therapy with antibiotics—for example, short- and long-term changes in the normal human microbiota, weight gain, gastroesophageal reflux disease and antibiotic resistance—have not been addressed in previous studies. In addition, before implementing large-scale programmes, further investigations are required regarding programme costs, feasibility, and appropriate target groups for the intervention, as well as region-specific data.

Several clinical trials that are currently underway should help clarify whether and how to implement population-based *H. pylori* screening and treatment programmes and should resolve many related uncertainties. The largest of these was initiated in 2011 in Linqu County in China with 184,786 residents aged 25–54 from a high-risk population [101]. *H. pylori*-positive participants ascertained by 13C-urea breath test were assigned to one of two groups receiving either a 10-day quadruple anti-*H. pylori* treatment or lookalike placebo together with a single dose of omeprazole and bismuth.

In the UK, the *H. pylori* Screening Study was initiated in 1997 to assess whether screening for and eradicating *H. pylori* infection in 56,000 healthy men aged 35–69 and women aged 45–69 would reduce the incidence of gastric cancer with a planned followup of 15 years or more after recruitment [102]. A multicentre randomised study to evaluate whether *H. pylori* screening followed by eradication in infected participants and endoscopy of those with serological evidence of atrophic gastritis could reduce gastric cancer mortality continues, recruiting in Latvia and potentially in neighbouring countries with the target number of 30,000 after completion of its pilot phase in 2016 [103]. In the Republic of Korea, a multicentre, double-blind randomised controlled trial to evaluate the preventive efficacy and effectiveness of *H. pylori* treatment on gastric cancer among 40–65 year-old participants recruited from the nationwide cancer screening programme was launched in 2014. The trial with a target number of 11,000 participants is scheduled to run for 10 years with biennial endoscopic followup within the screening programmes and populations [104].

Whilst there are concerns regarding increasing *H. pylori* antibiotic resistance [105] and other adverse events as noted above, standard *H. pylori* antibiotic eradication therapies have been evaluated in multiple clinical studies before their application in controlled trials to evaluate their impact on gastric cancer. To date, no other clinically validated approach for the eradication of *H. pylori* is currently available, despite extensive preclinical vaccine studies on preventative and therapeutic *H. pylori* vaccines [106,107]. Future strategies could investigate, for example, targeting CagA interactions with key cellular tumour suppressor proteins, such as the apoptosis-stimulating protein of p53-2 (ASPP2) [108]. CagA binding to ASPP2 blocks ASPP2 binding to the tumour suppressor p53 [108]. Modelling of CagA–ASPP2 interactions may facilitate the identification of small peptide inhibitors that could block CagA-induced deregulation of tumour suppressors [108].

### 5.2. Strategies for Eradication of H. pylori Infection and Their Evaluation

In December 2013, a Working Group meeting convened by IARC reviewed the evidence regarding eradication of *H. pylori* as a strategy for gastric cancer prevention [109]. The participants in the Working Group concluded that the high global burden of gastric cancer and the feasibility of treating its principal cause make it a logical target for intervention [110]. The Group also concluded, from a review of economic models, that population-based *H. pylori* screening and treatment would be both feasible and cost-effective in preventing gastric cancer [111]. The Group recommended that countries explore the possibility of introducing population-based *H. pylori* screening and treatment programmes but within the context of a scientifically valid assessment of programme processes, feasibility, effectiveness and possible adverse consequences [109,110]. The introduction in regions of high gastric cancer incidence of population-based *H. pylori* screening and treatment programmes, with a scientifically valid assessment of programme processes, feasibility, effectiveness and possible adverse consequences would affect the incidence of *H. pylori*-induced gastric cancer.

Given the recent molecular understanding of the oncogenic role of CagA, targeting *H. pylori* screening and treatment programmes in populations with a high incidence of *H. pylori* CagA-positive strains, particularly the more oncogenic East Asian *H. pylori* CagA strains, may be worth further exploration to optimise the benefits of such programmes.

## Figures and Tables

**Table 1:** Cancer registries with high and low age-standardised (world) incidence rates of gastric cancer, males, 2008–2012 (Source: Cancer Incidence in Five Continents, Volume XI adapted from [2], 2017, International Agency for Research on Cancer).

Registry	ASR	Registry	ASR
China, Yanting County	144.6	Jordan, Jordanians	5.0
Japan, Yamagata	77.4	USA Florida, White	4.6
Republic of Korea, Daejeon	68.8	Philippines, Rizal	4.2
India, Mizoram	47.2	Saudi Arabia, Riyadh, Saudi	3.5
Chile, Bio Bio	41.4	USA, Utah	3.4
Belarus	30.3	Thailand, Khon Kaen	3.0
Colombia, Pasto	26.5	India, Poona	3.0
Russian Federation, Samara	25.7	Malaysia, Penang, Malay	2.9
USA, Los Angeles, Korean	24.4	Kuwait	2.6
Costa Rica	20.9	South Africa, Eastern Cape	1.4

ASR, age-standardised rate per 100,000 person-years.

**Table 2:** Predicted gastric cancer burden 2012–2030 (Source: GLOBOCAN 2012, adapted from [1], 2013, International Agency for Research on Cancer).

Year	No. Gastric Cancers (Millions)	
	Demographic Effect	Demographic and −2.0% APC
2012	0.95	0.95
2015	1.03	0.97
2020	1.17	1.00
2025	1.34	1.03
2030	1.52	1.06
